# Antinociceptive Activity and Redox Profile of the Monoterpenes (+)-Camphene, *p*-Cymene, and Geranyl Acetate in Experimental Models

**DOI:** 10.1155/2013/459530

**Published:** 2013-01-14

**Authors:** Lucindo Quintans-Júnior, José C. F. Moreira, Matheus A. B. Pasquali, Soheyla M. S. Rabie, André S. Pires, Rafael Schröder, Thallita K. Rabelo, João P. A. Santos, Pollyana S. S. Lima, Sócrates C. H. Cavalcanti, Adriano A. S. Araújo, Jullyana S. S. Quintans, Daniel P. Gelain

**Affiliations:** ^1^Departamento de Bioquímica, Instituto de Ciências Básicas da Saúde, UFRGS, 90035-003 Porto Alegre, RS, Brazil; ^2^Departamento de Fisiologia, Universidade Federal de Sergipe (DFS/UFS), Aracaju, 49100-000 São Cristovão, SE, Brazil; ^3^Universidade Estadual de Feira de Santana (UEFS), 44031-460 Feira de Santana, BA, Brazil

## Abstract

*Objective*. To evaluate antinocicpetive and redox properties of the monoterpenes (+)-camphene, *p*-cymene, and geranyl acetate in *in vivo* and *in vitro* experimental models. *Methods*. Evaluation of the *in vitro* antioxidant activity of (+)-camphene, *p*-cymene, and geranyl acetate using different free radical-generating systems and evaluation of antinociceptive actions by acetic acid-induced writhing and formalin-induced nociception tests in mice. *Results*. *p*-Cymene has the strongest antinociceptive effect, but (+)-camphene and geranyl acetate also present significant activity at high doses (200 mg/kg). (+)-Camphene had the strongest antioxidant effect *in vitro* at TBARS and TRAP/TAR assays and also had the highest scavenging activities against different free radicals, such as hydroxyl and superoxide radicals. Sodium nitroprussiate-derived NO production was enhanced by (+)-camphene. Geranyl acetate and *p*-cymene also presented some antioxidant effects, but with a varying profile according the free radical-generating system studied. *Conclusion*. (+)-Camphene, *p*-cymene, and geranyl acetate may present pharmacological properties related to inflammation and pain-related processes, being potentially useful for development of new therapeutic strategies, with limited possibilities for *p*-cymene and geranyl acetate.

## 1. Introduction

In developing countries and/or areas inhabited by indigenous populations, plants and other natural sources constitute the solely source of bioactive molecules used for a variety of purposes. The use of medicinal plants throughout thousands of years by these populations allowed accumulation of empirical knowledge of their utility, which demands adequate evaluation of efficacy, safety, and action mechanisms [1]. The therapeutic properties of certain medicinal plants are generally related to their content of secondary metabolites, such as polyphenols, terpenes, phytosteroids, and alkaloyds, among others, which are produced in considerable amounts and variable proportions [2]. Essential oils are concentrated volatile aromatic compounds produced by aromatic plants, such as *Cymbopogon winterianus* Jowitt (Poaceae), *Cymbopogon citrates *Stapf (Poaceae), *Lavandula multifida* Linnaeus (Lamiaceae), and *Thymus pubescens* Boiss. Et Kotschy ex Celak (Lamiaceae) that have been found to exhibit a variety of biological properties [3–6]. Monoterpenes are the main chemical constituents of the essential oils of these plants and are found as mixtures of odoriferous components that can be obtained by steam distillation or solvent extraction from a large variety of aromatic plants. They are found in edible as well as in medicinal plants with a therapeutic property [7–10].

**Figure 1:**
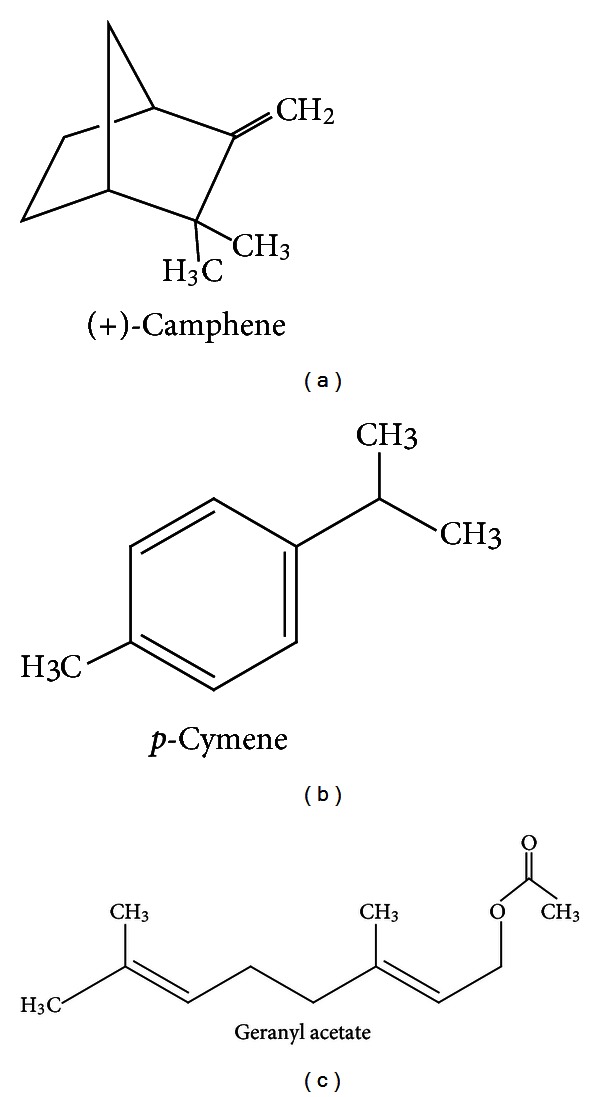
Chemical structure of (+)-camphene, *p*-cymene, and geranyl acetate.

Recent works have demonstrated that monoterpenes may present important pharmacological properties including antimicrobial [11], antioxidant [3], analgesic [12], and antitumoral [9] activities, as well as effects on cardiovascular system [13] and central nervous system (CNS) [14]. (+)-camphene, *p*-cymene, and geranyl acetate (Figure 1) are monoterpenes present in the essential oils of various plant species, such as Cypress, Origanum, and Eucalyptus oils [15, 16]. These substances are present at significant amounts in a wide variety of products derived from natural sources used as food, medicines, or other purposes in different countries. However, reports with reference to their therapeutic effects by studies aiming to establish their individual characteristics, as described in the present work, are scarce in literature.

Oxidative stress is the result of an unbalance in reactive species production and antioxidant defense and is a main component in cancer, infectious diseases, cardiovascular disorders, and neurodegenerative conditions [17]. Pharmacological agents showing therapeutic efficiency against some diseases may exert antioxidant properties in target tissues, which may be related to their mechanism of action [18]. Monoterpenes isolated from medicinal plants have been previously described as redox-active molecules, being able to scavenge specific reactive species such as hydroxyl radicals and nitric oxide (NO), preventing oxidation of biomolecules and influencing pain and inflammation [3, 19, 20]. Since reactive species and oxidative stress are linked to a wide array of pathological conditions, the evaluation of the redox action of monoterpenes with potential pharmacological activity may indicate new pharmacological agents for such diseases.

In the present work, we performed a screening of redox activities and antinociceptive actions of the monoterpenes (+)-camphene, *p*-cymene, and geranyl acetate. These monoterpenes are active compounds isolated from several medicinal plants traditionally used in Brazil and also in other countries to treat a wide range of chronic and/or infectious diseases related to pain, inflammation, and oxidative stress [13]. Studies with plant extracts or other products containing one or more of these substances have been conducted previously, but conclusions on the potential properties of their isolated constituents are highly limited due the presence of several metabolites in these preparations.

## 2. Material and Methods

### 2.1. Chemicals

Acetic acid, (+)-camphene (95% purity), *p*-cymene (≥97% purity), geranyl acetate (98% purity) (Figure 1), polyoxyethylene-sorbitan monolate (Tween 80), AAPH (2,2′-Azobis(2-methylpropionamidine)dihydrochloride), luminol (5-amino-2,3-dihydro-1,4-phthalazinedione), 2-deoxyribose, glycine, Griess reagent, SNP (sodium nitroprusside), TBA (2-thiobarbituric acid), (4,6-dihydroxypyrimidine-2-thiol), H_2_O_2_ (hydrogen peroxide), adrenaline, catalase, and SOD (superoxide dismutase) were purchased from Sigma (USA). Diazepam and aspirin were purchased from União Química (Brazil). All other reagents used in this study were of analytical or HPLC grade.

### 2.2. Animals

Adult male albino Swiss mice (28–34 g) were randomly housed in appropriate cages at 21 ± 2°C with a 12/12-h light/dark cycle (light from 06:00 to 18:00), with free access to food (Purina, Brazil) and tap water. We used 6–8 animals in each group. Nociceptive tests were carried out by the same visual observer and all efforts were made to minimize the number of animals used as well as any discomfort. Experimental protocols were approved by the Animal Care and Use Committee (CEPA/UFS no. 26/09) at the Federal University of Sergipe.

### 2.3. Acetic Acid-Induced Writhing

We followed the procedure by Koster et al. [21]. Mice (*n* = 8, per group) were pretreated either by (+)-camphene, *p*-cymene, or geranyl acetate (50, 100 or 200 mg/kg),   acetylsalicylic acid (Aspirin, 200 mg/kg), and the vehicle (saline + Tween-80 0.3%) by intraperitoneal route (i.p.). Then, after 1 h, mice received the 0.65% acetic acid injection (i.p., 0.25 mL/animal). Each animal was placed in an individual observation chamber, and 15 minutes after acetic acid injection the cumulative number of writhing responses was recorded for 15 minute after a latency period of 5 minutes.

### 2.4. Formalin-Induced Nociception

The procedure described by Hunskaar and Hole [22] was used. Nociception was induced by injecting 20 *μ*L of 1% formalin in distilled water in the right hind paw subplantar. Mice (*n* = 8, per group) previously received the same treatments described in the writhing test (1 h prior to injecting formalin). These mice were individually placed in a transparent plexiglass cage observation chamber (25 cm × 15 cm × 15 cm). The amount of time each animal spent licking the injected paw was indicative of pain. The number of lickings from 0–5 min (early phase) and 15–30 min (late phase) were counted after formalin injection. This volume and percentage concentration of formalin were selected from our pilot studies that showed a pain-related biphasic behavioral response (face-rubbing) of great intensity at periods of 0–5 minutes (first phase) and 15–40 minutes (second phase). Pain was quantified at those periods by measuring the time (in seconds) that the animals spent facerubbing in the injected area with their fore- or hindpaws.

### 2.5. Total Reactive Antioxidant Potential (TRAP) and Total Antioxidant Reactivity (TAR)

The total reactive antioxidant potential (TRAP) is employed to estimate the nonenzymatic antioxidant capacity of samples *in vitro*. This method is based on the quenching of luminol-enhanced chemiluminescence (CL) derived from the thermolysis of AAPH as the free radical source [23]. Briefly, we prepared AAPH solution, added luminol (AAPH + luminol, free radical-generating system) and then waited the system to stabilize for 2 h before the first reading. Different concentrations of each substance were added and the luminescence produced by the free radical reaction was quantified in a luminescence plate reader (Microbeta 1450, Perkin Elmer, Boston, MA, USA) during 60 min. The results were transformed in percentage and area under curve (AUC) and calculated in the GraphPad Prism version 5.01 software.

The total antioxidant reactivity (TAR) was also analyzed in the same samples used for TRAP readings. The TAR results were calculated as the ratio of light intensity in absence of samples (I^0^)/light intensity right after sample addition. Although TAR and TRAP evaluations are obtained in the same experiment, they represent different observations, since the TAR is more related to the antioxidant quality (reactivity, the scavenging capacity in a short-term period) and TRAP is more related to the antioxidant amount and kinetic behavior.

### 2.6. Hydroxyl Radical-Scavenging Activity

The formation of ^•^OH (hydroxyl radical) from Fenton reaction was assessed using the 2-deoxyribose oxidative degradation assay. The principle of the assay is the incubation of 2-deoxyribose with a hydroxyl radical generation system, which produces malondialdehyde (MDA). This system is then incubated with 2-thiobarbituric acid (TBA), which reacts with MDA and forms a chromophore quantifiable by spectrophotometry [24]. Briefly, typical reactions were started by the addition of Fe^2+^ (FeSO_4_ 6 *μ*M final concentration) to solutions containing 5 mM 2-deoxyribose, 100 mM H_2_O_2_, and 20 mM phosphate buffer (pH 7.2).

To measure the antioxidant of activity of each compound against hydroxyl radicals, different concentrations of (+)-camphene, *p*-cymene, and geranyl acetate were added to the system before Fe^2+^ addition. Reactions were carried out for 15 min at room temperature and were stopped by the addition of 4% phosphoric acid (v/v) followed by 1% TBA (w/v, in 50 mM NaOH). Solutions were boiled for 15 min at 95°C and then cooled at room temperature. The absorbance was measured at 532 nm and results were expressed as percentage of TBARS formed.

### 2.7. Nitric Oxide (NO^•^) Scavenging Activity

NO scavenging activity was quantified as previously described [25]. NO was generated from spontaneous decomposition of sodium nitroprusside (SNP) in 20 mM phosphate buffer (pH 7.4). Once generated, NO interacts with oxygen to produce nitrite ions, which were measured by the Griess reaction. The reaction mixture (1 mL) containing 10 mM SNP and either (+)-camphene, *p*-cymene, or geranyl acetate at different concentrations were incubated at 37°C for 1 h. An aliquot of 0.5 mL was taken and homogenized with 0.5 mL Griess reagent. The absorbance of chromophore was measured at 540 nm. Results were expressed as percentage of nitrite formed by SNP alone.

### 2.8. Thiobarbituric Acid-Reactive Species (TBARS)

Thiobarbituric acid-reactive substances (TBARS) assay was employed to quantify lipid peroxidation and an adapted TBARS method was used to measure the antioxidant capacity of the monoterpenes using egg yolk homogenate as lipid rich substrate [19]. The principle of the method is based on spectrophotometric measurement of the color produced during the reaction of thiobarbituric acid (TBA) with lipoperoxidation products, such as malondialdehyde and 4-hydroxynonenal. Briefly, egg yolk was homogenized (1% w/v) in 20 mM phosphate buffer (pH 7.4), 1 mL of homogenate was sonicated and then homogenized with 0.1 mL of (+)-camphene, *p*-cymene, or geranyl acetate at different concentrations. Lipid peroxidation was induced by addition of 0.1 mL of AAPH solution (0.12 M). Control was the incubation medium without AAPH. Reactions were carried out for 30 min at 37°C. Samples (0.5 mL) were centrifuged with 0.5 mL of TCA 15% at 1200 ×g for 10 min. An aliquot of 0.5 mL from the supernatant was mixed with 0.5 mL TBA (0.67%) and heated at 95°C for 30 min. After cooling, samples absorbance was measured using a spectrophotometer (UV-1800 Shimadzu) at 532 nm. The results were expressed as percentage of TBARS formed by AAPH alone “induced control”.

### 2.9. Superoxide-Dependent Adrenaline Autooxidation (“SOD-Like” Activity)

The ability of (+)-camphene, *p*-cymene, and geranyl acetate to scavenge superoxide anion (“superoxide dismutase-like” activity or “SOD-like” activity) was measured as previously described [26]. Samples were mixed with 200 *μ*L glycine buffer (50 mM, pH 10.2) and 5 *μ*L of native catalase 100 U/mL. Superoxide generation was initiated by addition of adrenaline 2 mM and adrenochrome formation was monitored at 480 nm for 5 minutes at 32°C. Superoxide production was determined by monitoring the reaction curves of samples and measured as percentage of the rate of adrenaline autooxidation into adrenochrome.

### 2.10. Catalase-Like Activity

The capacity of (+)-camphene, *p*-cymene, and geranyl acetate to degrade hydrogen peroxide (H_2_O_2_) added in the incubation medium (“catalase-like” or “CAT-like” activity) was measured as previously described [25]. Briefly, H_2_O_2_ diluted in 0.02 M phosphate buffer (pH 7.0), to obtain a 5 mM final concentration, was added to microplate wells, in which different concentrations of (+)-camphene, *p*-cymene, and geranyl acetate were present. Readings were made in a spectrophotometric plate reader (SpectraMax 190-Molecular Devices) at 240 nm every 15 seconds for 5 minutes at 37°C. Catalase-like activity was monitored based on the rate decomposition of H_2_O_2_. Data were expressed as percentage of the rate decomposition of H_2_O_2_.

### 2.11. Statistical Analysis

Data were evaluated using GraphPad Prism version 5.01 (Graph Pad Prism Software Inc., San Diego, CA, USA), through analysis of variance (ANOVA) followed by Tukey's test. The results are presented as mean ± SEM. In all cases, the differences were considered significant if *P* < 0.05.

## 3. Results

### 3.1. Acetic Acid-Induced Writhing

All tested doses of *p*-cymene produced significantly (*P* < 0.05 or *P* < 0.001) antinociceptive effect in this test compared to control group (vehicle) (Table 1). Pretreatment with (+)-camphene or geranyl acetate, at higher doses, significantly reduced nociceptive behavior compared with control group. Aspirin (200 mg/kg), used as positive control, also produced a significant antinociceptive effect (*P* < 0.001).

### 3.2. Formalin-Induced Pain

The highest doses of either (+)-camphene or geranyl acetate caused a significant inhibition of the licking response to the injected paw in mice, compared with the control group, only in the second phase of the formalin test (Table 1). However, *p*-cymene, in all doses, significantly inhibited (*P* < 0.01 or *P* < 0.001) both phases of formalin test when compared with control group. Additionally, *p*-cymene-treated mice were more significantly protected when compared with (+)-camphene or geranyl acetate-treated animals. As expected, aspirin (200 mg/kg) reduced the licking time only in second phase.

### 3.3. Rotarod Test

**Figure 2:**
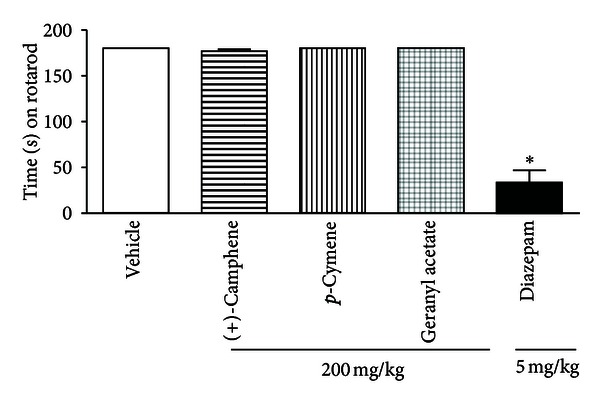
Time (s) on the rotarod observed in mice after i.p. treatments. The Statistical differences versus vehicle-treated mice group were calculated using ANOVA, followed by Tukey's test (*n* = 8, per group), *P < 0.001.

Monoterpene-treated mice did not show any significant motor performance alterations with the doses of 200 mg/kg (Figure 2). As might be expected, the CNS standard depressant diazepam (5 mg/kg, i.p.) reduced the time of treated animals on the rotarod apparatus (*P* < 0.001) compared with the control group.

### 3.4. Lipid Peroxidation

**Figure 3:**
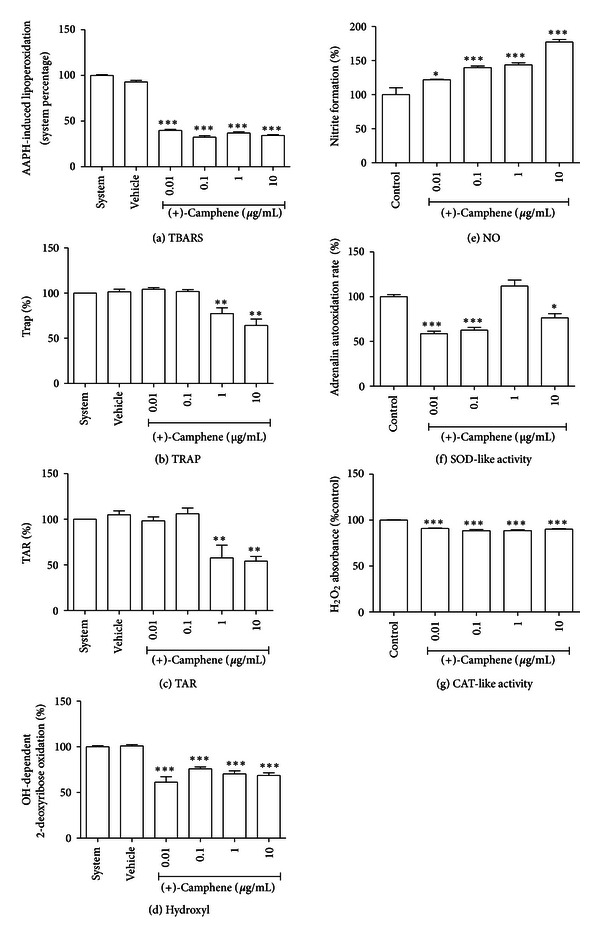
*In vitro* evaluation of the redox profile of (+)-camphene. (a) TBARS *in vitro* assay for lipid peroxidation assessment. (b) TRAP and (c) TAR values. (d) Hydroxyl radical-scavenging activity assay. (e) Nitric oxide (NO) scavenging assay. (f) Superoxide dismutase-like (SOD-like) activity. (g) Catalase-like (CAT-like) activity. Vehicle was DMSO 0.1% in all tests; in NO-scavenging activity, SOD-like and CAT-like activity tests, control is DMSO 0.1% alone. Bars represent mean ± SEM values. *P < 0.05, **P < 0.001, ***P < 0.0001 (1-way ANOVA followed by Tukey's *post-hoc* test).

**Figure 4:**
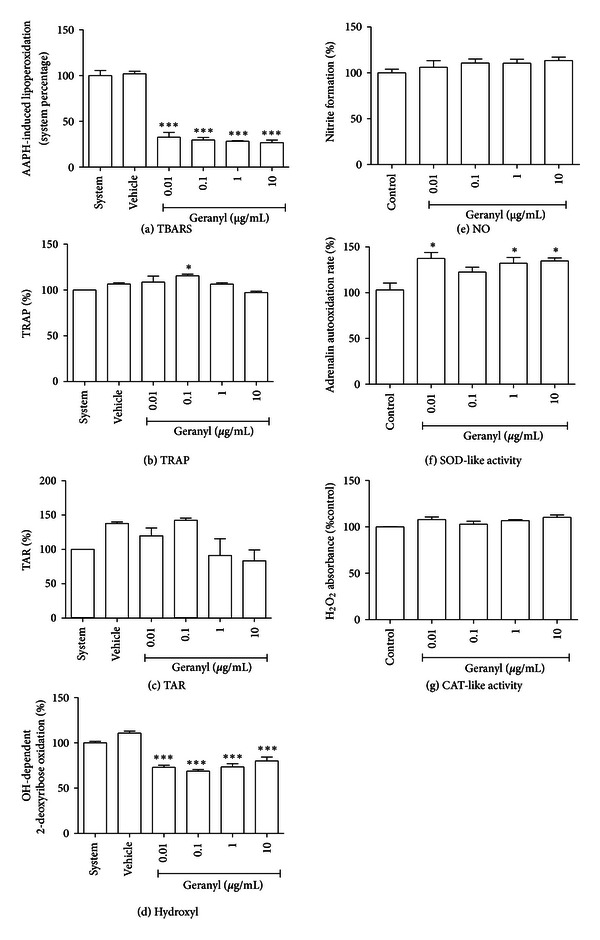
*In vitro* evaluation of the redox profile of geranyl acetate. (a) TBARS *in vitro* assay for lipid peroxidation assessment. (b) TRAP and (c) TAR values. (d) Hydroxyl radical-scavenging activity assay. (e) Nitric oxide (NO) scavenging assay. (f) Superoxide dismutase-like (SOD-like) activity. (g) Catalase-like (CAT-like) activity. Vehicle was DMSO 0.5% in all tests; in NO-scavenging activity, SOD-like, and CAT-like activity tests, control is DMSO 0.5% alone. Bars represent mean ± SEM values. *P < 0.05, **P < 0.001, ***P < 0.0001 (1-way ANOVA followed by Tukey's *post-hoc* test).

**Figure 5:**
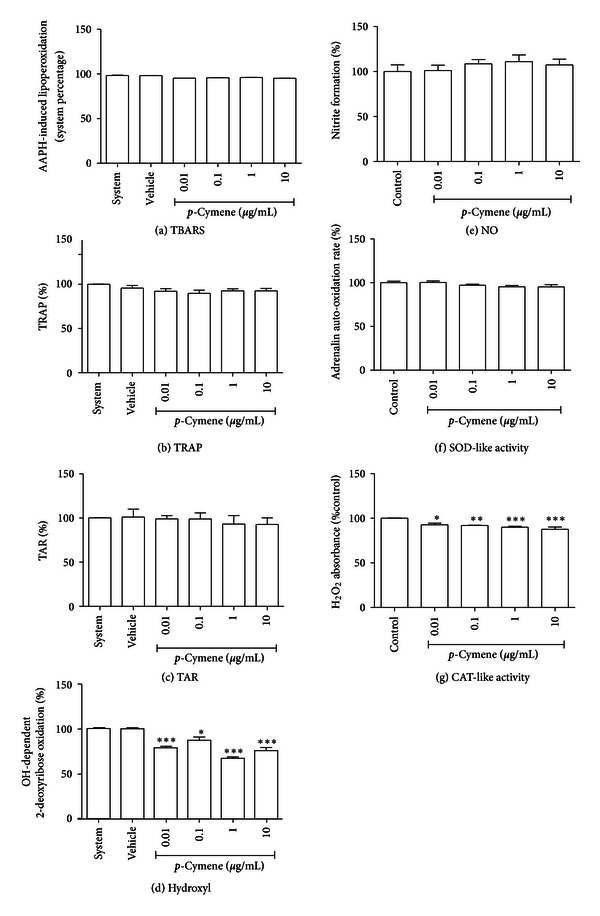
*In vitro* evaluation of the redox profile of *p*-cymene. (a) TBARS *in vitro* assay for lipid peroxidation assessment. (b) TRAP and (c) TAR values. (d) Hydroxyl radical-scavenging activity assay. (e) Nitric oxide (NO) scavenging assay. (f) Superoxide dismutase-like (SOD-like) activity. (g) Catalase-like (CAT-like) activity. Vehicle was DMSO 0.1% in all tests; in NO-scavenging activity, SOD-like and CAT-like activity tests, control is DMSO 0.1% alone. Bars represent mean ± SEM values. *P < 0.05, **P < 0.001, ***P < 0.0001 (1-way ANOVA followed by Tukey's *post-hoc* test).

To evaluate the antioxidant properties of three monoterpenes, we first assessed the ability of each compound to prevent lipid peroxidation in an *in vitro* peroxyl-generating system. (+)-camphene prevented lipoperoxidation induced by AAPH (Figure 3(a)). Geranyl acetate also presented antioxidant activity (Figure 4(a)). On the other hand, *p*-cymene had no effect on AAPH-induced lipoperoxidation (Figure 5(a)).

### 3.5. TRAP and TAR Parameters

To further explore the redox profile of these compounds, the TRAP/TAR parameters were evaluated, which indicate the capacity of a given sample to act as a general antioxidant or prooxidant agent in a constant reactive species generating system. We observed that (+)-camphene presents a significant antioxidant activity, which was indicated by both TRAP and TAR parameters (Figures 3(b) and 3(c)). On the other hand, geranyl acetate did not present antioxidant activity; in fact, a significant prooxidant effect was observed in the TRAP assay (Figures 4(b) and 4(c)). *p*-Cymene had no effect towards antioxidant or prooxidant activity in both TRAP and TAR measurements (Figures 5(b) and 5(c)).

### 3.6. Redox Activities against Isolated Free Radicals

(+)-camphene, geranyl acetate, and *p*-cymene all presented significant antioxidant activity against hydroxyl radicals generated *in vitro*, although at varying degrees (Figures 3(d), 4(d) and 5(d)). Besides, (+)-camphene presented a dose-dependent activity of NO generation in relation to control (Figure 3(e)), while geranyl acetate (Figure 4(e)) and *p*-cymene (Figure 5(e)) had no effect towards NO formation or scavenging activity. (+)-Camphene also presented increased SOD-like activity, indicating ability to scavenge or inhibit superoxide radicals (Figure 3(f)). Geranyl acetate, on the other hand, enhanced superoxide-mediated adrenaline oxidation (Figure 4(f)), while *p*-cymene had no statistically significant effect (Figure 5(f)). Finally, the CAT-like assay demonstrated that (+)-camphene (Figure 3(g)) and *p*-cymene (Figure 5(g)) have a modest, although significant, activity against H_2_O_2_, while geranyl acetate had no effect (Figure 4(g)).

## 4. Discussion

Our results showed that the monoterpenes here evaluated are able to inhibit the nociceptive behavior in mice, as determined by a significant reduction in acetic acid-induced abdominal writhing. Acetic acid-induced abdominal constriction is a standard, simple, and sensitive test for measuring analgesia induced by both central and peripherally acting analgesics [22, 27]. In acetic acid-induced abdominal writhing, pain is elicited by the injection of an irritant such as acetic acid into the peritoneal cavity, which produces episodes of characteristic stretching (writhing) movements, and inhibition of the number of episodes by analgesics is easily quantifiable [27]. To investigate if the treatments with (+)-camphene, *p*-cymene, or geranyl acetate could influence the motor activity of the animals and consequently impair the assessment of the nociceptive behavior in the experimental models, the motor activity of the animals was evaluated with a rotarod apparatus [28]. Monoterpenes-treated mice did not show any significant motor performance change when evaluated in rotarod test. Results showed in the present work support the hypothesis of (+)-camphene, *p*-cymene, and geranyl acetate participation in the inhibition of prostaglandin (PGE) synthesis, as the nociceptive mechanism involves the process or release of arachidonic acid metabolites via cyclooxygenase (COX) and PGE biosynthesis [29] during abdominal writhing induced by acetic acid.

The formalin test is a very useful method for not only assessing antinociceptive drugs but also helping in the elucidation of their action mechanisms. The neurogenic phase, commonly denominated first phase, is probably a direct result of paw stimulation and reflects centrally mediated pain with release of substance P while the late phase is due to the release of histamine, serotonin, bradykynin and prostaglandins [27]. Only *p*-cymene was able to reduce nociceptive behavior in both phases of the formalin test. The second phase, denominated “inflammatory phase,” depends on a combination of ongoing inputs from nociceptive afferents, due to the release of excitatory amino acids, PGE2, NO, tachykinin, and kinins among other peptides and, at least in part, of central sensitization [30, 31]. Additionally, intraplanar injection of formalin has been described to induce the production and release of NO [32], which in turn is suggested to be an essential component of the proinflammatory/nociceptive response by the stimulation of the production and release of cytokines, reactive species, and prostanoids [33]. On the other hand, it is generally agreed that N-methyl-D-aspartate (NMDA) receptors contribute to the persistent chemical stimulus during the late phase of central sensitization of dorsal horn neurons [34]. Our results show that monoterpenes produced an inhibition of the inflammatory pain, later phase, in mice as determined by a significant reduction of nociceptive behavior in second phase of formalin test.

Apparently (+)-camphene and geranyl acetate demonstrated a discrete analgesic profile when compared with *p*-cymene. In fact, some analgesic effects of *p*-cymene were previously demonstrated by our group [35]. We here compared this activity with other monoterpenes and attempted to establish a correlation of anti-inflammatory and analgesic effects of these compounds with their redox properties, as it is suggested for other monoterpenes [3]. Monoterpenes with oxygen in their structure constitute a wide group of antioxidant molecules, largely due to their functional groups (alcohols) [18].

We performed a detailed screening of the redox properties of these monoterpenes to establish their antioxidant properties, as reactive oxygen/nitrogen species (ROS/RNS) mediate inflammatory processes and are involved in the molecular mechanisms of several pathologies [36]. Terpenoids exhibiting antioxidant properties have been considered potential candidates for new therapeutic agents, especially when found in medicinal plants traditionally used to treat ROS/RNS-related diseases. Although *p*-cymene had a stronger antinociceptive effect, when compared to the other terpenes, this compound exhibited a poor antioxidant potential *in vitro*. *p*-Cymene did not prevent AAPH-induced lipoperoxidation, suggesting it is not able to act as a membrane antioxidant; besides, TRAP and TAR parameters did not show any effect of *p*-cymene on the *in vitro* free radical-generating system. High concentrations of *p*-cymene had a modest effect against H_2_O_2_ and hydroxyl radicals; however, it did not prevent NO and superoxide radicals formation, which are reactive species more related to pain and inflammation [36]. Hydroxyl radical has a high oxidant power and it is probably the most reactive radical [37]. It is able to join DNA nucleotides and cause strand breakage, which contributes to carcinogenesis, mutagenesis, and cytotoxicity. Nevertheless, (+)-camphene had a pronounced antioxidant effect at TBARS assay, which was confirmed at TRAP/TAR measurements, CAT-like activity and hydroxyl-scavenging assays. Furthermore, (+)-camphene presented significant superoxide degradation activity but enhanced NO formation. Geranyl acetate had a mixed redox profile, with antioxidant activity in TBARS and hydroxyl-scavenging assays and prooxidant activity at TRAP/TAR measurements and SOD-like activity assay, while the NO-scavenging assay showed no activity.

NO is a dual molecule playing major roles in both cell signaling and oxidative/nitrosative stress in a concentration and time-dependent manner [37, 38]. This molecule may be physiologically generated through NO synthases activity, triggering anti-inflammatory and cytoprotective pathways. However, NO also may increase the synthesis/release of proinflammatory mediators such as cytokines and reactive oxygen species [39] and prostanoids [33], thus promoting inflammatory reaction. We observed that (+)-camphene enhanced NO production *in vitro*, and our *in vivo* results show that (+)-camphene has a modest antinociceptive activity. Peripherally released NO contributes to the development of oedema and hyperalgesia in tissue injury and inflammation [36]. As mentioned above, expression of several inducible enzymes that contribute to the release of proinflammatory mediators such as NO and PGE2 are observed during inflammation, such as inducible nitric oxide synthase (iNOS) and cyclooxygenase-2 (COX-2). COX-2 is the inducible form of the enzyme, the synthesis of which is triggered by cytokines that also induce iNOS. The two pathways interact closely and NO can stimulate COX-2 activity by combining with its heme component [40].

The monoterpene *p*-cymene had no activity against NO, but presented the highest antinociceptive effect. Antioxidant properties are generally associated to “beneficial” effects, mainly due to the widespread association between free radicals with diseases and ageing. However, when a given substance presents little or none antioxidant activity at one or several *in vitro* assays, this may not be associated to lack of therapeutic properties. This apparent contradiction may rely on the fact that interaction of NO with other reactive species results in loss of its regulatory properties; for instance, NO-mediated activation of COX and subsequent release of beneficial and anti-inflammatory prostaglandins is lost when superoxide production is also enhanced, since interaction between superoxide and NO leads to *in situ* formation of peroxynitrite, a potent cytotoxic and proinflammatory reactive species [36, 37]. Although *p*-cymene had no effect against NO, we observed a mild activity against superoxide at SOD-like activity assay, which may attenuate NO deleterious effects and preserve its beneficial properties.

## 5. Conclusion

We show here a screening of antinociceptive actions and redox properties of three monoterpenes isolated from medicinal plants. Assays with animals demonstrated that *p*-cymene has the strongest antinociceptive effect, but (+)-camphene and geranyl acetate also present significant activity at higher doses. However, (+)-camphene had the strongest antioxidant effect at TBARS and TRAP/TAR assays, and also had the highest scavenging activities against different free radicals generated by *in vitro* systems. Geranyl acetate and *p*-cymene also presented some antioxidant effect, but with a varying profile according the free radical-generating system studied. The results presented here suggest that (+)-camphene, *p*-cymene, and geranyl acetate may present pharmacological properties related to inflammation and pain-related processes, being potentially useful for development of new therapeutic strategies, with limited possibilities for *p*-cymene and geranyl acetate.

## Figures and Tables

**Table 1:** Effect of (+)-camphene, *p*-cymene, geranyl acetate, or aspirin on writhing induced by acetic acid and formalin-induced nociception tests.

Treatment	Dose (mg/kg)	Writhing test	Formalin test	
		Number of writhings^a^	0–5 min^a^	15–30 min^a^
Vehicle	—	27.8 ± 3.1	85.7 ± 8.8	113.8 ± 28.6
(+)-Camphene	50	22.5 ± 5.3	76.1 ± 9.6	107.1 ± 11.2
(+)-Camphene	100	25.1 ± 4.7	81.7 ± 8.3	68.3 ± 14.7^b^
(+)-Camphene	200	15.7 ± 4.4^c^	72.4 ± 12.8	44.3 ± 11.9^c^
*p*-Cymene	50	8.9 ± 5.9^c^	43.5 ± 7.1^b,&,#^	49.4 ± 9.5^c^
*p*-Cymene	100	4.1 ± 0.9^d,&,#^	24.0 ± 8.9^d,&,#^	33.5 ± 10.0^d^
*p*-Cymene	200	1.3 ± 0.5^d,&,#^	11.9 ± 5.1^d,&,#^	25.4 ± 7.4^d,&,#^
Geranyl acetate	50	23.9 ± 5.7	77.0 ± 11.4	98.7 ± 16.6
Geranyl acetate	100	13.0 ± 4.8^c^	65.9 ± 13.2	55.4 ± 9.1^c^
Geranyl acetate	200	15.7 ± 3.6^c^	79.9 ± 10.3	59.3 ± 11.2^c^
Aspirin	200	5.1 ± 3.2^d^	78.3 ± 17.8	27.5 ± 11.1^d^

*n* = 8, per group.

^a^Values represent mean ± SEM.

^b^ *P* < 0.05, ^c^ *P* < 0.01 or ^d^ *P* < 0.001 (one-way ANOVA and Turkey's post hoc test), significantly different from control group.

^&^ *P* < 0.05 or *P* < 0.01 (one-way ANOVA and Turkey's post hoc test), significantly different from (+)-camphene-treated group.

^#^ *P* < 0.05 or *P* < 0.01 (one-way ANOVA and Turkey's post hoc test), significantly different from geranyl acetate-treated group.
